# Investigation of microorganisms in cannabis after heating in a commercial vaporizer

**DOI:** 10.3389/fcimb.2022.1051272

**Published:** 2023-01-13

**Authors:** Danielle S. Sopovski, Jing Han, Marla Stevens-Riley, Qiang Wang, Bruce D. Erickson, Berk Oktem, Michelle Vanlandingham, Cassandra L. Taylor, Steven L. Foley

**Affiliations:** ^1^ Division of Microbiology, Food and Drug Administration (FDA) National Center for Toxicological Research, Jefferson, AR, United States; ^2^ Office of Pharmaceutical Quality, Food and Drug Administration (FDA) Center for Drug Evaluation and Research, Silver Spring, MD, United States; ^3^ Office of Science and Engineering Laboratories, Food and Drug Administration (FDA) Center for Devices and Radiological Health, Silver Spring, MD, United States; ^4^ Division of Biochemical Toxicology, Food and Drug Administration (FDA) National Center for Toxicological Research, Jefferson, AR, United States

**Keywords:** cannabis, microbial populations, metagenomics, microbial culture, vaporizer

## Abstract

**Introduction:**

There are concerns about microorganisms present on cannabis materials used in clinical settings by individuals whose health status is already compromised and are likely more susceptible to opportunistic infections from microbial populations present on the materials. Most concerning is administration by inhalation where cannabis plant material is heated in a vaporizer, aerosolized, and inhaled to receive the bioactive ingredients. Heating to high temperatures is known to kill microorganisms including bacteria and fungi; however, microbial death is dependent upon exposure time and temperature. It is unknown whether the heating of cannabis at temperatures and times designated by a commercial vaporizer utilized in clinical settings will significantly decrease the microbial loads in cannabis plant material.

**Methods:**

To assess this question, bulk cannabis plant material supplied by National Institute on Drug Abuse (NIDA) was used to assess the impact of heating by a commercial vaporizer. Initial method development studies using a cannabis placebo spiked with *Escherichia coli* were performed to optimize culture and recovery parameters. Subsequent studies were carried out using the cannabis placebo, low delta-9 tetrahydrocannabinol (THC) potency and high THC potency cannabis materials exposed to either no heat or heating for 30 or 70 seconds at 190°C. Phosphate-buffered saline was added to the samples and the samples agitated to suspend the microorganism. Microbial growth after no heat or heating was evaluated by plating on growth media and determining the total aerobic microbial counts and total yeast and mold counts.

**Results and discussion:**

Overall, while there were trends of reductions in microbial counts with heating, these reductions were not statistically significant, indicating that heating using standard vaporization parameters of 70 seconds at 190°C may not eliminate the existing microbial bioburden, including any opportunistic pathogens. When cultured organisms were identified by DNA sequence analyses, several fungal and bacterial taxa were detected in the different products that have been associated with opportunistic infections or allergic reactions including *Enterobacteriaceae*, *Staphylococcus, Pseudomonas*, and *Aspergillus*.

## Introduction

Cannabis (*Cannabis sativa* L.) is a plant of the Cannabaceae family and contains more than eighty biologically active chemical compounds (Food and Drug Administration, 2020b). Parts of the *Cannabis sativa* L. plant have been controlled under the Controlled Substances Act (CSA) since 1970 under the drug class “Marihuana” (commonly referred to as “marijuana”) [21 U.S.C. 802(16)] (Food and Drug Administration, 2021a; Office of the Law Revision Counsel, 2022; Drug Enforcement Agency, 2022). The Agriculture Improvement Act of 2018, Pub. L. 115-334 (the 2018 Farm Bill), removed “hemp” from the CSA, which means that cannabis plants and derivative that contain no more than 0.3 percent on a dry weight basis are no longer controlled substances under federal law. Additionally, there are a variety of state laws permitting or decriminalizing the use of cannabis in 37 states as of 2022 (Seltenrich, 2019; MPP, 2022b). An estimated 5.5 million individuals are registered in various state-level medical programs (MPP, 2022a). In addition, there is interest in studying cannabis for several different health conditions including, but not limited to, Parkinson’s disease; mental health disorders such as Schizophrenia, anxiety and depression; chronic pain; and for HIV and cancer patients to increase appetite and decrease nausea and vomiting (Whiting et al., 2015; Sexton et al., 2016); however, there are major concerns about microbial contamination in cannabis and the potential for infections since some users may be significantly immunocompromised (Ruchlemer et al., 2015). There have been multiple reported cases of infections associated with cannabis use caused by fungi and bacteria in immunocompromised individuals using inhaled cannabis material (Taylor et al., 1982; Verweij et al., 2000; Benedict et al., 2020). Even as the state-level cannabis laws have rapidly developed, there is no consistency among states and the standards are highly variable across different states. Additionally, there is no federally adopted standards for microbial testing of these products as these products remain illegal at the federal level (Seltenrich, 2019; Pruyn et al., 2022).

As part of the evaluation of Investigational New Drug (IND) applications proposing to utilize cannabis for therapeutic indications in patients, the FDA must consider the safety of the product that is being proposed (Food and Drug Administration, 2020b). When used in clinical trials, any investigational drug product must meet FDA’s requirements and standards for drug products under 21 CFR part 312 (Food and Drug Administration, 2021b), and this includes microbiological quality. However, testing of botanical raw material for microorganisms is challenging. Cannabis products are heterogeneous mixtures of plant material, and their bioactive constituents and microbial profiles can vary greatly due to agriculture practices, growing and storage conditions, and processing procedures. These variables can lead to a diversity of bioactive characteristics and potentially varying levels of microbial populations in different products (Holmes et al., 2015; Ward, 2018). A common delivery mechanism for cannabis materials is through the use of vaporization to volatilize the bioactive compounds for potential therapeutic purposes (National Institute on Drug Abuse, 2020; Lim et al., 2022). Because heat treatment is a common way to kill microorganisms, the question on whether the heating conditions utilized in commercial vaporizers (to volatilize bioactive compounds) impacts microbial populations is of interest. Key taxa of concern for cannabis are fungi such as *Aspergillus*, *Mucor*, and *Penicillium* and bacteria including *Clostridium*, *Streptococcus, Salmonella*, and certain *Escherichia* (Holmes et al., 2015).

There has been an increase in commercial testing platforms available for microbial testing of cannabis (Boyar et al., 2021; Ward, 2018). Most methods rely on either culture-based or PCR-based approaches that are extensions of approaches used for foods or botanical products (Holmes et al., 2015; Ward, 2018). While culturing has limitations, it has been the standard for microbial testing and is used to quantify microorganisms in cannabis products (Boyar et al., 2021). Recently FDA issued a draft Guidance for Industry entitled “Cannabis and Cannabis-Derived Compounds: Quality Considerations for Clinical Research” that reflects FDA’s current thinking related to clinical research and the manufacturing of drug products derived from cannabis and cannabis-derived compounds (Food and Drug Administration, 2020a). As part of the draft guidance, the recommendation is to follow the compendium of microbiological methods presented in the United States Pharmacopeia (USP) Chapters <61> “*Microbiological Examination of Nonsterile Products: Microbial Enumeration Tests*” and <62> “*Microbiological Examination of Nonsterile Products: Tests for Specified Microorganisms*” (U. S. Pharmacopeial Convention, 2019b; U. S. Pharmacopeial Convention, 2019a). However, metagenomics and DNA sequencing of bacterial and fungal DNA present in cannabis products is becoming more widespread because they provide culture-independent determinations of bacteria and fungi present and their relative proportions within a product (McKernan et al., 2016; Boyar et al., 2021). In the current study we utilize both culture and metagenomics-based approaches to evaluate impact of heating on microbial populations in cannabis products.

## Material and methods

### Cannabis test materials

Dried cannabis plant materials were supplied as unfiltered cigarettes by the National Institute on Drug Abuse (NIDA) and were grown at the University of Mississippi. Three types of cannabis material were utilized in the studies: placebo without measurable THC, low potency containing 1.90% delta-9 THC, and high potency containing 6.50% delta-9 THC (**Table 1**). The placebo was prepared by ethanol extraction of cannabis material. To prepare the cannabis material for the experiments, ~200 cigarettes from each type were aseptically opened, the paper was removed, the material was mixed to homogenize, and the final mixture was stored at -20°C until use. Prior to individual experiments, the cannabis material was humidified overnight at room temperature by placing in plastic weigh boats within separate lab desiccators containing 25 mL saturated NaCl and maintained at room temperature.

**Table 1:** Characteristics of the cannabis materials used in the study.

	NIDA Batch ID*	Delta-9 THC Level	CBD Level	Initial TMAC Growth	Date of Production
**Placebo**	12944-0509-105	0.00%	0.00%	None Detected	May, 2009
**Low potency**	12792-1208-77	2.00%	0.02%	2.18 X 10^6^ CFU/g	December, 2008
**High potency**	10604-0203-95	6.70%	None Detected	1.76 X 10^6^ CFU/g	February, 2003

*https://nida.nih.gov/research/research-data-measures-resources/nida-drug-supply-program-dsp/nida-drug-supply-program-dsp-ordering-guidelines/marijuana-plant-material-available.

### Bacterial strain

*Escherichia coli* BL-21 (Invitrogen) was used for cannabis spiking experiments. The culture was removed from -80°C storage, streaked onto a tryptic soy agar (TSA) with 5% sheep’s blood and incubated at 37°C overnight. Colonies were used to inoculate Luria-Bertani (LB) broth, which was incubated at 37°C overnight. The next morning, the optical density was measured spectrophotometrically at 600nm (Genesys 20, ThermoFisher) to estimate the cell numbers and the cell concentrations adjusted to the desired level with DPBS and placed at 4°C for spiking experiments.

### Cannabis material sample preparation

Sample preparation for microbial testing was based on methods recommended in USP Chapter <61> (U. S. Pharmacopeial Convention, 2019a). Initially, 10 grams each of cannabis placebo, low potency, and high potency materials that had been homogenized, thawed, and humidified, were placed on one side of a Whirl-Pak filter bag (Nasco) and 90 ml of Dulbecco’s phosphate-buffered saline (DPBS; pH 7.2) was added to suspend the material. The bags were sealed and shaken at 150 rpm for 15 minutes at 20°C to dislodge the microorganisms. The bags were squeezed to push the microbial suspension through a filter to separate the filtrate from cannabis material. The microbial filtrate was removed and serial dilutions up to 1:10,000 were prepared in DPBS for plating. However, after these initial experiments, the amount of cannabis material used in all subsequent studies was reduced to 70 mg to be consistent with the volume to fill the sample holder in the vaporizer.

### Evaluation of recovery methods

Humidified placebo (70 mg each) was spiked with either 20 µl of prepared *E. coli* suspension of approximately 5 x 10^8^ CFU/ml (OD_600_ of ~1.0) in DPBS or 20 µl of sterile DPBS and aseptically mixed and stored on ice for 2 hours. Additionally, 20 µl samples of the *E. coli* suspension were added to tubes and processed to serve as a reference to determine the efficiency of the microbial recovery techniques. After incubation, 4 ml of DPBS was added to each sample and the tubes were rotated for 15 min at room temperature. Serial dilutions were prepared and 50 µl was plated in triplicate onto TSA plates. The plates were incubated at 37°C overnight and counted. To calculate the efficiency of recovery, the number of *E. coli* cultured from the cannabis materials was divided by the number of *E. coli* that grew from the reference suspensions. The ratio was then multiplied by 100 to determine the percent recovery from each sample. Each set of samples was prepared in triplicate and the experiments were done three times (total of 27 counts for each sample type: un-spiked control, spiked sample, and reference *E. coli* suspension).

### Vaporization equipment and heating method development

A commercial vaporizer, Volcano Digit (Storz & Bickel GmbH & Company) was used to assess the impact of heating on microbial levels. The vaporizer was turned on to heat up for 5 minutes prior to sample introduction. Based on manufacturer use recommendations for volatilization of the bioactive compounds in plant materials, a temperature of 190°C was used throughout the study. To optimize testing conditions for the impact of heating, initial testing was conducted on the cannabis placebo spiked with 20 µl of the *E. coli* suspension (5 x 10^8^ CFU/ml) as described above. This initial spiking volume was found to be too large for the 70 mg samples when added to the sample holder in the vaporizer, so spike volumes were reduced to 5 µl with an ~8 x 10^8^ CFU/ml (OD_600_ of ~1.025) suspension. The spiked cannabis material was placed in the vaporizer sample holder and subjected to 1 of 3 conditions: no heating, heating for a 30-second preheating cycle (recommended time prior to attachment of the vapor collection bag) or heating for 70-seconds (representing sample preheating and the 40 seconds needed for filling the vaporizer bag for typical use). Each set of samples contained three replicates that were plated in triplicate and the experiments were done a total of four times (total of 36 counts for each sample type: no heat, 30 seconds of heating, and 70 seconds of heating). To assess the impact of heating on the spiked samples, the numbers of *E. coli* isolated from the heated cannabis samples were compared to those isolated from the no heat samples to determine the relative reductions in recoveries.

### Impact of heating on microbial counts

Placebo, low and high potency cannabis materials were exposed to no heat, 30 seconds, or 70 seconds of heating in the Volcano vaporizer. To each sample, 4 ml of DPBS was added and they samples were processed and plated on TSA as described above to determine aerobic microbial levels. Additionally, the suspensions were plated onto Sabouraud dextrose agar (SDA) and potato dextrose agar (PDA) plates to assess fungal growth. The SDA and PDA plates were incubated at 25°C for up to 5 days and the resulting colonies were counted to determine the impact of heating on fungal populations. Plating was done in triplicate and the experiments repeated a total of three times (experiments 1-3).

### Microbial metagenomics studies

To further characterize the microorganisms in the sample, metagenomics experiments were conducted on the samples utilized in experiment 3. These efforts consisted of two sets of sequencing experiments. The first examined the bacterial and fungal populations that were present in the microbial suspensions from the cannabis materials prior to plating (i.e., the DPBS used to extract the microorganisms for the cannabis materials). The second set of sequencing focused on the identification of microorganisms that grew following plating. To prepare the DNA to conduct the sequencing for the first set of experiments, the undiluted microbial suspensions in DPBS from six representative subsamples each of placebo, low potency and high potency were added to the lysis tubes from the ZR Fungal/Bacterial DNA MiniPrep™ kit (Zymo Research) for template DNA extraction. For the second set of experiments, growth from six representative tryptic soy agar (TSA) and six Sabouraud dextrose agar (SDA) plates from each cannabis material type were collected by scraping the surface of the plates with an inoculating loop and suspending the growth in the dilution buffer from the ZR Fungal/Bacterial DNA MiniPrep™ kit. The DNA was then extracted from each sample according to manufacturer’s instructions (Zymo Research). The isolated DNA served as template to amplify the 16s rRNA V3/V4 or internal transcribed spacer (ITS) variable regions. The 16s rRNA sequencing was conducted following the methods described by Caporaso et al. (2011) using the following primers TCGTCGGCAGCGTCAGATGTGTATAAGAGACAGXXXXXXACTCCTACGGGNGGCWGCAG (forward); and GTCTCGTGGGCTCGGAGATGTGTATAAGAGACAGGACTACHVGGGTATCTAATCC (reverse). For ITS sequencing, the primers were TCGTCGGCAGCGTCAGATGTGTATAAGAGACAGXXXXXXCTTGGTCATTTAGAGGAAGTAA (forward) and GTCTCGTGGGCTCGGAGATGTGTATAAGAGACAGTCCTCCGCTTATTGATATGC (reverse) and the reactions carried out as described previously (Seifert, 2009). The XXXXXX in the forward primers indicates the location of the sample-specific barcodes. The amplified and indexed DNA was purified using QIAquick PCR Purification Kit (Qiagen) and quantified using a Nanodrop (ThermoFisher) and Qubit fluorimeter (ThermoFisher). Equimolar amounts of purified PCR products were pooled for an Illumina MiSeq sequencing run using a 2 x 250 or 2 x 300 reaction kit (Illumina). The microbiome bioinformatics were performed using QIIME2 (2021.2) (Bolyen et al., 2019). Raw sequence data were demultiplexed using the q2-demux plugin and paired-end sequences were joined, quality filtered, and denoised with DADA2 (Callahan et al., 2016). Bacterial 16S reads were classified using preformatted SILVA 138 99% reference sequence and taxonomy files (Quast et al., 2013; Bokulich et al., 2018; Bokulich and Parker, 2021). ITS sequences (forward reads) were classified using the QIIME release version 8.3 from UNITE (Abarenkov et al., 2021). For plated samples, the analyses were qualitative (OTUs present) vs. quantitative (relative numbers of each OTU), while for diluent samples relative proportions of taxa, β diversity was calculated using the Bray-Curtis dissimilarity method.

## Results and discussion

Heating and subsequent vaporization of bioactive cannabis compounds followed by inhalation by individuals is a predominant method of cannabis use (National Institute on Drug Abuse, 2020; Lim et al., 2022). Thus, the aim of the study was to gain a better understanding of the impact of heating on microorganisms in cannabis material placed in vaporizers. Initial investigations to determine the microbial levels in all samples showed that 10 g samples from the placebo had no growth on TSA, while 2.18 X 10^6^ CFU/g were recovered from the low potency and 1.76 X 10^6^ CFU/g were recovered from the high potency samples (**Table 1**). Additionally, when reduced sample sizes (70 mg) were used, rarely (~8%) was any microbial growth detected among the plated placebo replicates and those with growth had a single colony. With this minimal microbial background, the placebo was chosen for the spiking experiments to optimize and assess the microbial recovery from cannabis materials. This lack of microorganisms limited enumeration challenges that may occur if there was a high resident microbial population to account for. *E. coli* was used for spiking experiments since it is a marker for fecal contamination and may be encountered in outdoor growing settings and with product handling (Thunberg et al., 2002; Simpson et al., 2002). The comparison of the amount of *E. coli* recovered from the spiked product versus the *E. coli* reference suspension used for the spiking showed that the recovery was 101.7% (SD: 20.4%) of the spiked amount, indicating that the recovery technique was efficient for extracting microorganisms for the cannabis materials.

**Figure 1:**
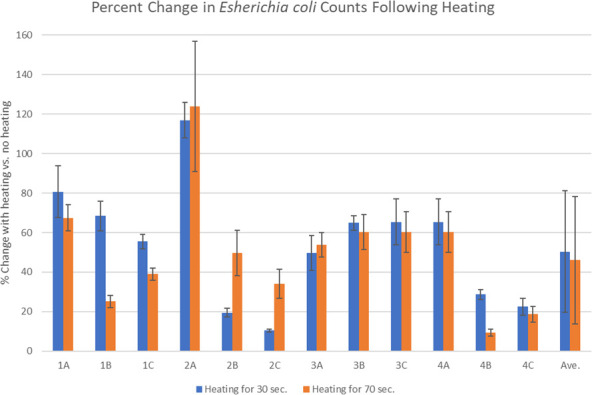
Percent change in the placebo following spiking with *E. coli* and treating with heat in the Volcano vaporizer for either 30 or 70 seconds. Each set of samples contained three replicates (A–C) that were plated in triplicate and the experiments were done a total of four times (1-4). The error bars indicate the standard deviation.

Once methods were developed to efficiently recover the spiked *E. coli*, the experiments focused on investigating the impact of heating on microbial populations. **Figure 1** shows the recoveries of *E. coli* from spiked placebo that was heated for 30 seconds and 70 seconds. The average reduction after heating for 30 seconds (preheating cycle) was 50.4% and after heating for 70 seconds (preheating, plus typical time to fill the vaporizer bag) was 46.1%. There was some experiment-to-experiment variability, especially in the second experiment where the first replicate appeared to have more *E. coli* following treatment and the other two replicates showing significant reductions (samples 2A, 2B and 2C in **Figure 1**).

**Figure 2:**
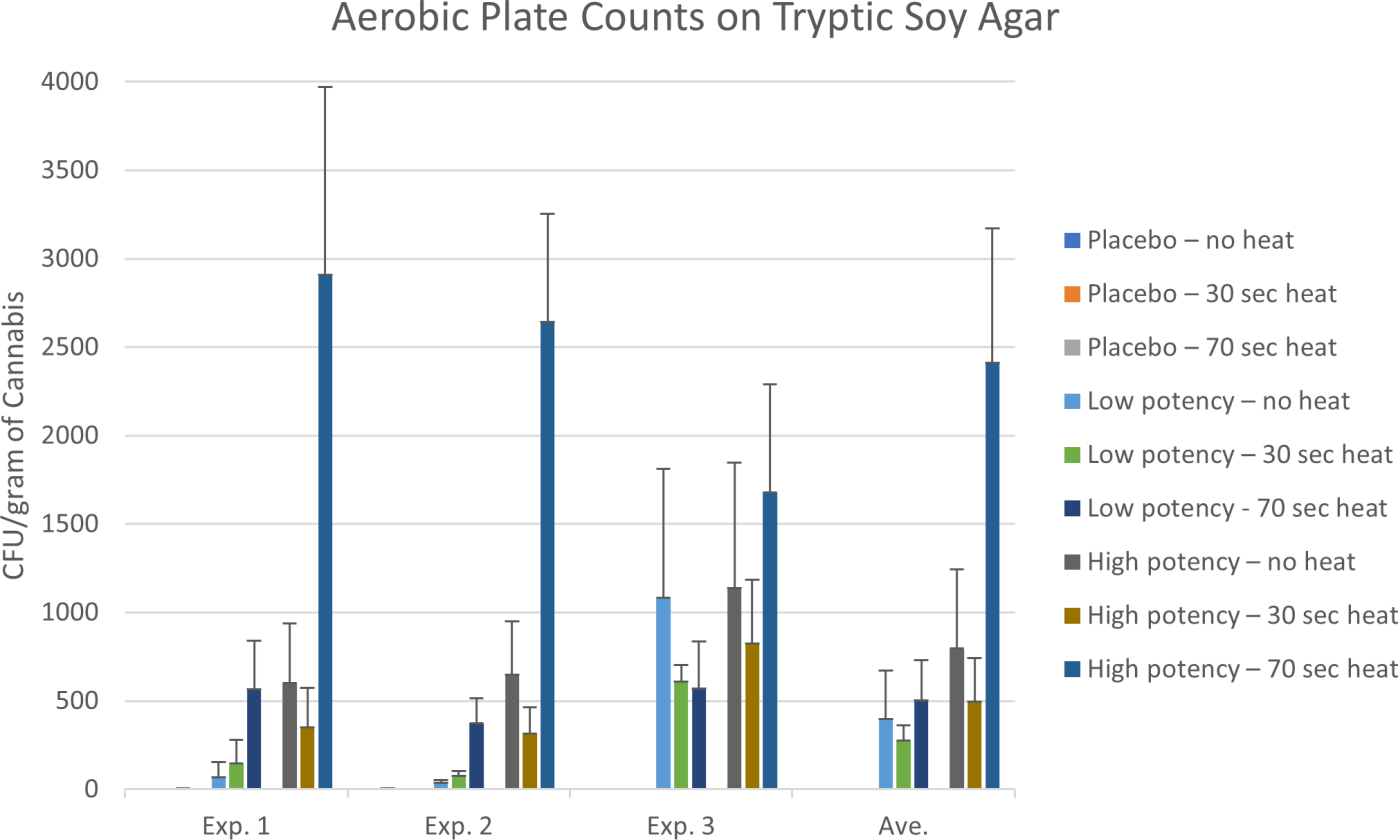
Aerobic microbial counts of the placebo, low potency, and high potency cannabis materials that were either not exposed to heating or heat for either 30 or 70 seconds in the Volcano vaporizer. The plates were counted after an 18-hour incubation. The error bars indicate the standard deviation.

**Figure 3:**
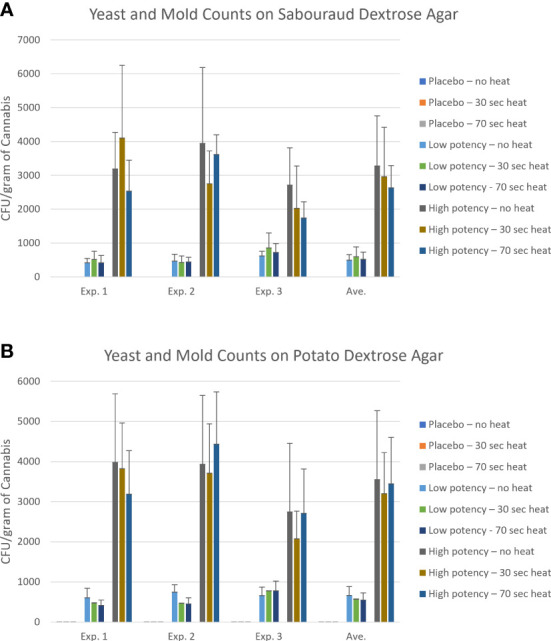
Yeast and mold counts of the placebo, low potency, and high potency cannabis materials that were either not exposed to heating or heat for either 30 or 70 seconds in the Volcano vaporizer. Samples were plated onto Sabouraud dextrose agar (panel **A**) or potato dextrose agar (panel **B**). The plates were counted after an incubation of up to 5 days. The error bars indicate the standard deviation.

**Tables 2** and **3** show the genera of bacteria and fungi that were isolated from the cannabis samples with different potencies and their presence before or after heating during the final set of experiments examining the impact of heating on microbial counts. Quantitative results of the impact of heating on the resident microbial populations of placebo, low potency, and high potency samples are shown in **Figures 2** and **3**. Overall, few bacteria or fungi were cultured from the placebo samples, regardless of whether heated or not. Bacterial genera identified in the placebo included *Bacillus, Staphylococcus* and *Acinetobacter.* For low potency samples, there was some variability across the experiments in the number of colonies on the TSA plates. For example, in experiments 1 and 2 examining the impact of heating on microbial counts, there was an increasing trend in the number of organisms detected with increasing heat exposure times. In experiment 3, the results were the opposite, indicating that there was a reduction in the number of organisms detected with heating. These findings may be an aberration driven by variability in the no-heat samples in experiment 3, that displayed intraexperimental variability, with one set of replicates matching the results of experiments 1 and 2. Bacteria identified included *Microbacterium, Bacillus, Terribacillus, Enterobacter, Kosakonia, Pantoea, Siccibacter*, and *Pseudomonas*. For high potency samples, trends in microbial numbers on TSA plates were consistent across all experiments, with a drop in numbers at 30 seconds, but an increase at 70 seconds (**Figure 2**). Bacteria identified in high potency samples included *Enterococcus, Enterobacter, Kosakonia, Pantoea, Siccibacter, Pseudomonas*, and *Stenotrophomonas* species. When the taxonomy data was viewed across the sample types, all bacterial taxa, with the exception of *Staphylococcus*, that were identified in at least one no-heat sample, were also collected from a sample following heating (**Table 2**). These data are indicative that heating under the conditions used were generally not sufficient to kill the bacteria present in the products.

**Table 2:** Bacterial taxa identified through 16s rRNA metagenomic sequence analyses of bacterial colonies cultured on tryptic soy agar plates.

Genus	Placebo	Low Potency	High Potency	No Heat	Heated
*Microbacterium*		X			X
*Bacillus*	X	X		X	X
*Terribacillus*		X		X	X
*Enterococcus*			X		X
*Staphylococcus*	X			X	
*Rhizobium*			X		X
*Enterobacterales* (order)	X	X		X	X
*Enterobacteriaceae* (family)	X	X	X	X	X
*Enterobacter*		X	X	X	X
*Kosakonia*		X	X	X	X
*Pantoea*		X	X		X
*Siccibacter*		X	X	X	X
*Acinetobacter*	X				X
*Pseudomonas*		X	X	X	X
*Stenotrophomonas*			X		X

an “X” indicates that the taxon was detected in at least one replicate from the particular sample type or heat treatment. These results were aggregated since the microbial growth was collected from plates where the samples were diluted for counting. The aggregation minimizes the potential underrepresentation of the taxa present in a sample type.

**Table 3:** Fungal taxa identified through ITS metagenomic sequence analyses of fungal colonies cultured on Sabouraud dextrose agar plates.

Genus	Placebo	Low Potency	High Potency	No Heat	Heated
*Cladosporium*	X	X		X	X
*Septoria*		X		X	X
*Didymellaceae* (family)		X		X	X
*Didymella*		X		X	X
*Epicoccum*		X			X
*Alternaria*	X	X	X	X	X
*Aspergillus*	X			X	X
*Talaromyces*	X			X	
*Diaporthe*			X		X
*Lectera*		X	X	X	X
*Gibberella*	X	X	X	X	X
*Paramyrothecium*		X			X
*Irpex*					X
*Bullera*		X		X	
*Hannaella*		X			X

an “X” indicates that the taxon was detected in at least one replicate from the particular sample type or heat treatment. These results were aggregated since the fungal growth was collected from plates where the samples were diluted for counting. The aggregation minimizes the potential underrepresentation of the taxa present in a sample type.

**Table 4** shows the bacterial taxa detected in the bulk material (pre-plating suspension) from each sample type, while **Supplemental Figure S1** shows the principal coordinates analysis (PCoA) plots of the β diversity analysis for the samples. Analysis of the β diversity of the microbial populations present in the cannabis materials showed that the samples from different potency materials tended to group together, regardless of whether they were exposed to heating or not (**Supplemental Figure S1A**). DNA from bacterial taxa including *Pantoea* and *Pseudomonas* were more abundantly detected in the low and high potency samples and *Pedobacter, Psychrobacillus, Paenibacillus*, and *Xanthomonas* more abundant in the placebo (**Table 4** and **Supplemental Table S1**). Isolates from the genus *Pantoea* and *Pseudomonas* were also identified among the cultured organisms from low and high potency samples (**Table 2**). Conversely, the more abundant taxa detected in the placebo subsamples (*Pedobacter, Psychrobacillus, Paenibacillus*, and *Xanthomonas*) were not cultured from the placebo; however, *Acinetobacter* species which was cultured from the samples accounted for about 2.5% of sequencing reads (i.e. sequencing output) from the placebo. Likewise, *Bacillus* species and members of the *Enterobacteriaceae* family which were also cultured also were detected in the sequencing results of the placebo samples, although they demonstrated low abundance with less than 0.1% of sequencing reads (**Supplemental Table S1**). The fact that the taxa with the highest numbers of sequencing reads from the cannabis samples were not cultured, likely indicated that they were killed during the preparation of the placebo material, yet their DNA remained present in the products.

**Table 4:** Relative percentage of bacterial taxa identified in each of the different sample types through 16s rRNA sequence analyses of the microbial suspensions prior to plating.

Genus	Placebo	Low Potency	High Potency
*Acinetobacter*	2.9	0.1	< 0.1
*Alkanindiges*	0.2	< 0.1	< 0.1
*Allorhizobium-Neorhizobium-Pararhizobium-Rhizobium*	0.3	< 0.1	2.3
*Aquabacterium*	0.1	< 0.1	< 0.1
*Arthrobacter*	4.3	0	< 0.1
*Aureimonas*	0.2	< 0.1	< 0.1
*Azospirillum*	0.1	0	< 0.1
*Blastococcus*	0.2	0	0
*Brevundimonas*	2.5	< 0.1	0.1
*Caulobacter*	0.4	< 0.1	< 0.1
*Caulobacteraceae* (Family)	0.1	< 0.1	< 0.1
*Chloroplast*	2.0	0.3	0.1
*Chthonobacter*	0.1	< 0.1	< 0.1
*Comamonadaceae* (Family)	0.6	< 0.1	< 0.1
*Comamonas*	0.3	< 0.1	< 0.1
*Cupriavidus*	0.2	< 0.1	< 0.1
*Cutibacterium*	0.3	< 0.1	< 0.1
*Devosia*	0.1	< 0.1	< 0.1
*Enhydrobacter*	0.1	< 0.1	< 0.1
*Enterobacteriaceae* (Family)	0	< 0.1	0.2
*Erwiniaceae* (Family)	0	< 0.1	0.3
*Escherichia-Shigella*	0.1	0	0
*Haemophilus*	0.1	0	0
*Kosakonia*	0.4	< 0.1	< 0.1
*Lysobacter*	0.2	< 0.1	< 0.1
*Marinilactibacillus*	0.2	< 0.1	< 0.1
*Massilia*	3.3	< 0.1	1.0
*Noviherbaspirillum*	0.6	< 0.1	< 0.1
*Novosphingobium*	< 0.1	< 0.1	0.7
*Paenibacillus*	17.7	< 0.1	0
*Pannonibacter*	0.2	< 0.1	< 0.1
*Pantoea*	0.6	86.21	50.9
*Paracoccus*	0.2	< 0.1	< 0.1
*Pedobacter*	8.5	< 0.1	< 0.1
*Phenylobacterium*	0.1	< 0.1	< 0.1
*Pigmentiphaga*	0	0	0.3
*Pseudomonas*	1.2	12.95	39.7
*Psychrobacillus*	30.2	< 0.1	0
*Qipengyuania*	0.1	0	< 0.1
*Rheinheimera*	0.2	< 0.1	< 0.1
*Rhizobiaceae* (Family)	0.2	0	< 0.1
*Rubellimicrobium*	0.1	< 0.1	< 0.1
*Sanguibacter*	2.3	0	0
*Serratia*	0.1	0	0
*Shewanella*	0.2	< 0.1	< 0.1
*Sphingomonas*	0.4	< 0.1	< 0.1
*Stenotrophomonas*	3.2	< 0.1	2.0
*Verticiella*	0.1	< 0.1	1.4
*Xanthomonas*	12.9	< 0.1	0.2

Note, table includes taxa with at least 0.1% of overall reads.

Samples highlighted in red were identified in greater than 10% of the reads from their respective sample type.

Our metagenomics analyses are consistent with analyses of others. McKernan and colleagues also used both metagenomics and culture-based approaches to assess microbial populations in cannabis materials (McKernan et al., 2016; McKernan et al., 2021) and detected several species of medical importance including *Acinetobacter baumannii, E. coli, Pseudomonas aeruginosa, Salmonella, Staphylococcus aureus*, and *Streptococcus pneumoniae*. When they sequenced DNA from bacterial colonies isolated on agar plates they identified *Pseudomonas, Bacillus, Enterobacter* and *Escherichia* strains (McKernan et al., 2021). Our findings identified similar taxa, including *Acinetobacter, Escherichia, Pseudomonas, Staphylococcus, Bacillus, Enterobacter, Enterococcus* and *Stenotrophomonas* identified in cannabis material either from colonies growing on agar plates or in the pre-plating suspension (**Tables 2** and **4**, respectively). Members of these genera have been associated with human infections, which is a concern because people who are most susceptible to infections are those with weakened immune systems and such individuals may use cannabis in an effort to relieve effects associated with their preexisting health condition, such as cancer chemotherapy or HIV (Sexton et al., 2016; Rosenthal and Pipitone, 2020). As cannabis is often used as an inhaled product, it is notable that members of genera including *Acinetobacter, Bacillus, Pseudomonas* and *Stenotrophomonas*, have been associated with causing opportunistic respiratory infections including pneumonia (Hartzell et al., 2007; Brooke, 2012; Shimoyama et al., 2017; Kumar et al., 2018; Ishida et al., 2019).

For the experiments examining fungal growth, samples were plated on SDA (**Figure 3A**) and PDA (**Figure 3B**). The placebo samples had very low counts, averaging <1 CFU/g on SDA, and fungi identified from the placebo samples included members of the genus *Cladosporium, Alternaria, Aspergillus, Irpex, Talaromyces* and *Gibberella*. On the PDA media, there was an observed, though not significant, increase in fungi recovered from the placebo following heating (0.30 CFU/g in no heat samples vs. 1.22 CFU/g in 70-second heated samples). For low potency samples, there was variability in results across the experiments. On the SDA media, the most common result was a slight increase in fungal numbers with 30-second heating, followed by a reduction in 70-second samples to levels close to no-heat levels (**Figure 3A**). Fungal genera identified in low potency samples included *Cladosporium, Septoria, Didymella, Epicoccum, Alternaria, Lectera, Gibberella, Paramyrothecium, Bullera* and *Hannaella*. For the PDA media, the general trend was reduction in the total counts with heating (experiment 3 was an exception, **Figure 3B**). High potency samples had overall higher fungal counts and more interexperimental variability in counts on SDA media. *Alternaria, Diaporthe, Lectera*, and *Gibberella* were detected in these samples. The general trend with the high potency samples seemed to indicate a reduction in fungi with heating, although there were exceptions (**Figure 3A**). When plated on PDA, there was a consistent reduction with the 30-second heating and with experiments 1 and 3 there was a reduction with 70-second heating too (**Figure 3B**). For all types of cannabis materials, the overall differences observed were not significant, indicating that heating did not effectively reduce total fungal numbers in the cannabis materials. When the fungal taxonomy data was assessed among the different sample types and heating conditions, only two genera, *Talaromyces* and *Bullera*, were found in at least one of the no-heat samples, but not in any of the samples following heating (**Table 3**).

With few exceptions the viable fungi identified on a culture plate (**Table 3**), were also detected in the microbial suspensions that were used for the dilutions and subsequent plating. One notable exception is with members of the genus *Filobasidium* which were detected in pre-plating suspension but did not appear to be cultured on any of the samples tested; thus, these members of this taxa may not have been viable in the subsamples where its DNA was isolated. **Table 5** provides an overview of the fungi that were detected in each of the samples and the PCoA plots displaying the β diversity of the samples is shown in **Supplemental Figure S1B**. When a taxon was identified on the culture plates, but not in the pre-plating suspension, there was typically a closely related (same family) taxon detected in the suspension. These apparent observed discrepancies may be due to challenges in analyses of metagenomic data that rely on matching to microbial databases and slight variations in the sequencing that can lead to some inconsistency in the identifications, such that the same organism sequenced in separate experiments being identified as closely related organisms. Overall, the taxa identified on the culture plates appeared to represent well what was detected in the suspensions used for the plating. Detailed results of the ITS sequencing experiments in provided in **Supplemental Table S2**.

**Table 5:** Relative percentage of fungal taxa identified in each of the different sample types through ITS sequence analyses of the microbial suspensions prior to plating.

Genus	Placebo	Low Potency	High Potency
*Acremonium*	0.4	0.2	2.2
*Alternaria*	34.7	9.7	19.3
*Aspergillus*	1.0	0.1	0.2
*Basidiomycota* (Phylum)	< 0.1	0.7	0.3
*Candida*	0	0.3	0
*Capnodiales* (Order)	0	0	0.3
*Catabotrydaceae* (Family)	0.7	0	0
*Cladosporium*	6.9	6.4	4.9
*Coprinellus*	0	0.1	0
*Coprinopsis*	0	0	0.5
*Curvularia*	0	0.1	0.2
*Cystobasidium*	< 0.1	0.6	1.0
*Diaporthe*	0	< 0.1	0.9
*Didymella*	0	0.4	0
*Didymellaceae* (Family)	0	0	1.9
*Dothideales* (Order)	0.4	0	0
*Epicoccum*	0.6	0	0
*Exserohilum*	0.2	0	0
*Filobasidium*	0.2	42.3	11.0
*Fungi* (Kingdom)	4.0	2.8	2.8
*Fusarium*	0	18.4	0
*Gibberella*	14.1	2.7	5.0
*Gibellulopsis*	0	0	0.2
*Hannaella*	< 0.1	0.2	22.0
*Hyphodontia*	0	0	0.4
*Kabatiella*	0	1.8	0.3
*Kwoniella*	0	0	0.7
*Lectera*	0	0.6	0
*Malassezia*	7.4	4.5	2.8
*Malasseziaceae* (Family)	0.1	0	0
*Moesziomyces*	0	0.1	< 0.1
*Mycosphaerella*	0	< 0.1	0.7
*Naganishia*	0	0.1	0.5
*Neodidymelliopsis*	0	0.1	0
*Nigrospora*	0	0	0.2
*Occultifur*	0	0.2	0
*Papiliotrema*	0	0	0.3
*Paradictyoarthrinium*	0.8	0	0
*Penicillium*	5.0	0.8	1.8
*Phallus*	0.1	0	0
*Physodontia*	0.3	0	0
*Plectosphaerella*	0	0	0.7
*Podosphaera*	0	0	0.1
*Pseudogymnoascus*	16.7	0.2	0
*Rhodotorula*	0	< 0.1	0.4
*Septoria*	0	0.1	0
*Sordariomycetes* (Class)	0	0.3	0
*Stachybotrys*	0.2	0	0
*Symmetrospora*	< 0.1	3.5	14.5
*Thanatephorus*	0	0	0.3
*Toxicocladosporium*	0	0.3	< 0.1
*Tremellomycetes* (Class)	0	0	3.3
*Vishniacozyma*	5.3	1.7	0.2
*Wallemia*	0	0.2	0
*Wickerhamomyces*	0.7	0	0

Note, table includes taxa with at least 0.1% of overall reads.

Samples highlighted in red were identified in greater than 10% of the reads from their respective sample type.

Fungi, including members of the genus *Aspergillus*, are a concern in inhaled products including cannabis and tobacco products (Verweij et al., 2000; Pauly and Paszkiewicz, 2011; Remington et al., 2015). Several aspergilli have been associated with allergic-type reaction and infections, which are a major concern for immunocompromised patients (Stevens and Melikian, 2011; Ruchlemer et al., 2015; Benedict et al., 2020). Fungi are also a concern due to the ability of certain species to produce mycotoxins including aflatoxins, ochratoxin A, patulin, and sterignatocystin (Varma et al., 1991; Pauly and Paszkiewicz, 2011; McKernan et al., 2015). Aflatoxin B1 can lead to an increased inflammatory response in the respiratory system and potentially lead to other pathologies (Pauly and Paszkiewicz, 2011). We detected members of the genus *Aspergillus* in samples, including after 70-second heating. Most of the other fungal taxa identified through the sequencing experiments (**Tables 3** and **Supplemental Table S2**) are plant-associated fungi; however, some have been associated with causing allergic reactions and/or infections in highly immunocompromised individuals, thus they may be a concern if inhaled (Yilmaz et al., 2014; Gabriel et al., 2016; Caillaud et al., 2021; Anees-Hill et al., 2021).

An early challenge of the project was optimizing microbial recovery techniques. Our initial efforts were done following the general methodologies described USP <61> methods (U. S. Pharmacopeial Convention, 2019a), which is the compendial chapter for determining microbial counts for non-sterile products used in human drug products. A challenge with this method, is the recommended large sample volume (10 g), which proved impractical in the evaluation of cannabis material used in a vaporizer that holds only approximately 70 mg of sample. Therefore, for most experiments we utilized a smaller 70 mg sample of product that is consistent with the amount that fits in the vaporizer sample holder. This change was not without issues; we initially reduced the DPBS volume to 0.63 ml (maintaining the original w/v ratio), but the volume was too low for efficient solubilization of microorganisms from the cannabis material. The DPBS volume was subsequently increased to 4 ml which greatly improved extraction of bacteria during *E. coli* spiking experiments and was used in all of the subsequent studies. The high potency samples were particularly difficult to suspend and extract the microorganisms due to the stickiness of the samples from the resin-like compounds. Both low and high potency materials tended to clump together and stick to tubes and pipet tips, which may have led to some variability observed in the recovery experiments. Our research team found similar challenges with smokeless tobacco products, which had a range of moisture and viscosity traits (Han et al., 2016). The increased diluent volumes employed helped to minimize many of these challenges.

The studies described were foundational, but they do have some limitations. Only a limited number of cannabis plant material samples were tested; however, the samples had a range of characteristics that provided valuable data on the impact of heating on microbial levels. The sequencing studies were performed on samples from the final experiment examining the impact of heating on microbial counts after observing a diversity of colony morphologies in the first two experiments that warranted a more in-depth characterization of the microbial populations. The sequencing data from individual TSA and SDA culture plates were aggregated for each cannabis type and heating status in **Tables 2** and **3** to provide a more comprehensive picture of the organisms present in each sample and whether those taxa were present following the heating process. With the limited exceptions noted above, bacteria and fungi taxa grew following heating. It should be noted that even though some taxa were not detected following heating, it does not necessarily indicate that heating specifically killed or inactivated these organisms, as they may have been diluted out prior to plating.

Overall, the study demonstrated that heating of the cannabis materials at 190°C in the vaporizer for 70 seconds (settings suggested by the manufacturer to volatize the bioactive compounds) did not lead to significant reductions in microorganisms present in the cannabis materials and in some cases, the heating seemed to enhance the numbers of organisms recovered. This latter observation may be due to the heat volatilizing some residual compounds that may have facilitated “sticking” of microorganisms to the plant material. Finally, it is unknown if the vaporizer airflow could facilitate the transfer of microorganisms from the cannabis material into the vapor collection bag that the end-user inhales. However, the findings demonstrate that heating with a vaporizer may not be considered an effective means to limit microbial hazards associated with inhaled cannabis use. Therefore, other microbial reduction methodologies, such as gamma irradiation, ultraviolet germicidal irradiation or pasteurization may be needed to limit microbial hazards in cannabis material (Hazekamp, 2016; Boyar et al., 2021).

## Data availability statement

The sequence data for this study have been deposited in the European Nucleotide Archive (ENA) at EMBL-EBI under accession number PRJEB58799 (https://www.ebi.ac.uk/ena/browser/view/PRJEB58799).

## Author contributions

CT, BO, MS-R and SF initiated the studies and provided leadership and overview for the studies. CT secured the funding for the research from the FDA Center for Drug Evaluation and Research. DS, JH, MV, BE, QW and SF conducted the studies. SF, CT, MS-R, DS, and BE conducted data analyses and interpretation. SF, CT, MS-R, and BE drafted the manuscript and all listed authors contributed to final editing and revisions of manuscript.
