# Supplementary material for: Investigation of microorganisms in cannabis after heating in a commercial vaporizer
Source: Front Cell Infect Microbiol. 2023 Jan 13;12:1051272. doi: 10.3389/fcimb.2022.1051272 (PMC9880168; doi:10.3389/fcimb.2022.1051272)
Supplement: Supplementary file 1 [file Image_1.pdf]

A

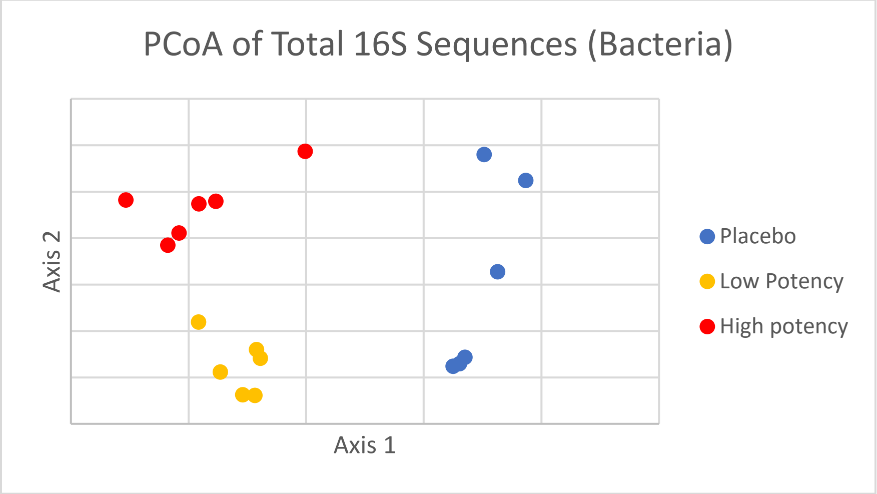

B

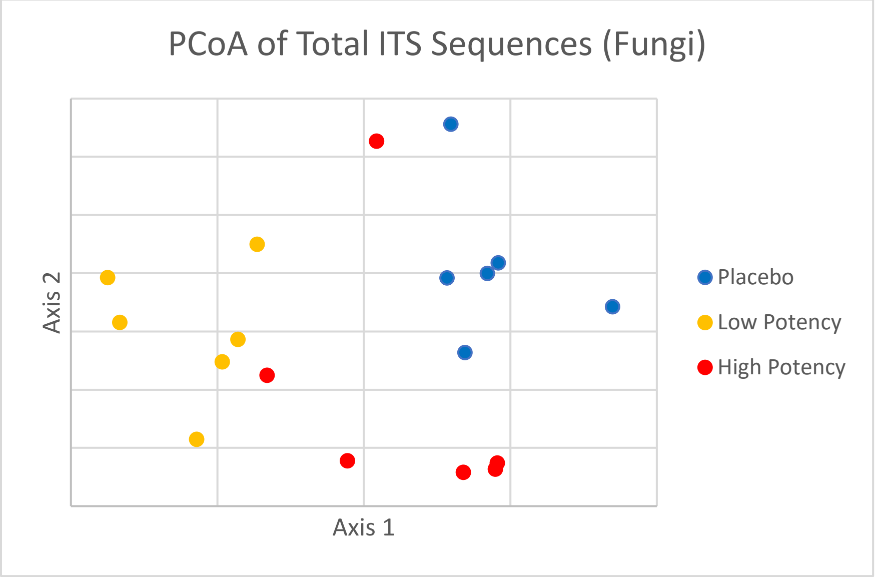

Figure S1. A) β-diversity plots of the bacterial taxa present in the placebo (gold), low potency (blue), and high potency (red) cannabis materials. **B)** β-diversity plots of the fungal taxa present in the placebo (gold), low potency (blue), and high potency (red) cannabis materials. In general, the samples grouped by sample type based on the populations regardless of whether or not they were exposed to heating in the Volcano vaporizer.
