# Supplementary material for: Investigation of microorganisms in cannabis after heating in a commercial vaporizer
Source: Front Cell Infect Microbiol. 2023 Jan 13;12:1051272. doi: 10.3389/fcimb.2022.1051272 (PMC9880168; doi:10.3389/fcimb.2022.1051272)
Supplement: Supplementary file 2 [file Table_1.pdf]

#OTU ID	P_0	P_0	P_30	P_30	P_70	P_70	L_0	L_0	L_30	L_30	L_70	L_70	H_0	H_0	H_30	H_30	H_70	H_70
p__Actinobacteriota;c__Actinobacteria;o__Actinomycetales;f__Actinomycetaceae;g__Actinomyces	0	0	0	0	0	90	0	0	0	0	0	0	0	0	0	0	0	0
p__Actinobacteriota;c__Actinobacteria;o__Corynebacteriales;f__Corynebacteriaceae;g__Corynebacterium	96	8	0	0	5	75	0	0	0	0	0	0	0	0	0	0	0	0
p__Actinobacteriota;c__Actinobacteria;o__Corynebacteriales;f__Segniliparaceae;g__uncultured	0	0	0	0	3	0	0	0	0	0	0	0	0	0	0	0	0	0
p__Actinobacteriota;c__Actinobacteria;o__Frankiales;f__Geodermatophilaceae;g__Blastococcus	23	24	6	0	24	223	0	0	0	0	0	0	0	0	0	0	0	0
p__Actinobacteriota;c__Actinobacteria;o__Frankiales;f__Geodermatophilaceae;g__Geodermatophilus	0	0	0	0	0	31	0	0	0	0	0	0	0	0	0	0	0	0
p__Actinobacteriota;c__Actinobacteria;o__Kineosporiales;f__Kineosporiaceae;g__Kineosporia	0	0	0	0	0	0	0	0	0	0	0	0	158	0	0	0	0	0
p__Actinobacteriota;c__Actinobacteria;o__Micrococcales;f__Beutenbergiaceae;g__Salana	0	0	0	0	0	0	0	0	0	0	0	0	0	0	0	0	0	59
p__Actinobacteriota;c__Actinobacteria;o__Micrococcales;f__Dermabacteraceae;g__Brachybacterium	4	0	0	0	0	0	0	0	0	0	0	0	0	0	0	0	0	0
p__Actinobacteriota;c__Actinobacteria;o__Micrococcales;f__Intrasporangiaceae;__	0	0	0	0	11	0	0	0	0	0	0	0	0	0	0	0	0	0
p__Actinobacteriota;c__Actinobacteria;o__Micrococcales;f__Microbacteriaceae;g__Agrococcus	0	0	0	0	0	0	0	0	0	10	0	0	0	0	0	0	0	0
p__Actinobacteriota;c__Actinobacteria;o__Micrococcales;f__Microbacteriaceae;g__Amnibacterium	0	0	0	0	0	0	0	0	0	0	0	0	10	0	0	0	0	0
p__Actinobacteriota;c__Actinobacteria;o__Micrococcales;f__Microbacteriaceae;g__Curtobacterium	0	0	0	0	0	0	25	0	5	0	0	0	0	0	0	0	0	20
p__Actinobacteriota;c__Actinobacteria;o__Micrococcales;f__Microbacteriaceae;g__Microbacterium	0	0	0	0	0	16	0	0	0	0	0	0	8	0	0	0	0	0
p__Actinobacteriota;c__Actinobacteria;o__Micrococcales;f__Microbacteriaceae;g__Mycetocola	0	0	4	0	0	0	0	0	0	0	0	0	0	0	0	0	0	0
p__Actinobacteriota;c__Actinobacteria;o__Micrococcales;f__Microbacteriaceae;g__Rathayibacter	0	0	0	0	4	0	0	0	0	0	0	0	0	0	0	0	0	0
p__Actinobacteriota;c__Actinobacteria;o__Micrococcales;f__Micrococcaceae;g__Arthrobacter	0	0	73863	0	0	6	0	0	0	0	0	0	0	0	6	0	0	0
p__Actinobacteriota;c__Actinobacteria;o__Micrococcales;f__Micrococcaceae;g__Kocuria	25	0	0	0	0	0	0	0	0	0	0	0	0	0	0	0	0	0
p__Actinobacteriota;c__Actinobacteria;o__Micrococcales;f__Micrococcaceae;g__Rothia	0	0	0	0	0	84	0	0	0	0	0	0	0	0	0	0	0	0
p__Actinobacteriota;c__Actinobacteria;o__Micrococcales;f__Sanguibacteraceae;g__Sanguibacter	0	0	40431	0	0	0	0	0	0	0	0	0	0	0	0	0	0	0
p__Actinobacteriota;c__Actinobacteria;o__Propionibacteriales;f__Nocardioidaceae;g__Nocardioides	6	12	0	0	6	0	0	0	0	0	0	0	0	0	0	0	0	0
p__Actinobacteriota;c__Actinobacteria;o__Propionibacteriales;f__Propionibacteriaceae;g__Cutibacterium	14	22	0	0	10	342	7	8	8	16	11	18	13	10	12	9	7	11
p__Actinobacteriota;c__Actinobacteria;o__Pseudonocardiales;f__Pseudonocardiaceae;g__Saccharopolyspora	0	0	0	0	0	95	0	0	0	0	0	0	0	0	0	0	0	0
p__Actinobacteriota;c__Thermoleophilia;o__Solirubrobacterales;f__Solirubrobacteraceae;g__Patulibacter	0	0	0	0	0	0	0	0	0	0	0	0	3	0	0	0	0	12
p__Armatimonadota;c__Armatimonadia;o__Armatimonadales;f__Armatimonadales;g__Armatimonadales	0	0	0	0	0	0	0	0	0	0	0	0	14	0	0	0	0	0
p__Bacteroidota;c__Bacteroidia;o__Bacteroidales;f__Porphyromonadaceae;g__Porphyromonas	0	0	0	0	0	0	0	0	0	2	0	0	0	0	3	0	5	0
p__Bacteroidota;c__Bacteroidia;o__Chitinophagales;f__Chitinophagaceae;g__Cnuella	0	0	0	0	0	0	0	0	0	0	0	0	0	0	0	6	0	0
p__Bacteroidota;c__Bacteroidia;o__Chitinophagales;f__Chitinophagaceae;g__Ferruginibacter	0	0	0	0	0	35	0	0	0	0	0	0	0	0	0	0	0	0
p__Bacteroidota;c__Bacteroidia;o__Chitinophagales;f__Chitinophagaceae;g__Flaviaesturariibacter	0	0	0	0	0	0	0	0	0	0	0	0	0	0	0	7	0	0
p__Bacteroidota;c__Bacteroidia;o__Chitinophagales;f__Chitinophagaceae;g__Parasegetibacter	0	2	0	0	0	0	0	0	0	0	0	0	0	0	0	0	0	0
p__Bacteroidota;c__Bacteroidia;o__Chitinophagales;f__Chitinophagaceae;g__Sediminibacterium	0	4	0	0	0	0	0	0	0	0	0	0	0	0	0	0	0	0
p__Bacteroidota;c__Bacteroidia;o__Chitinophagales;f__Chitinophagaceae;g__Terrimonas	0	0	0	0	0	0	0	0	0	0	0	0	3	0	0	0	0	0
p__Bacteroidota;c__Bacteroidia;o__Cytophagales;f__Cyclobacteriaceae;g__uncultured	0	4	0	0	0	0	0	0	0	0	0	0	0	0	0	0	0	0
p__Bacteroidota;c__Bacteroidia;o__Flavobacteriales;f__Flavobacteriaceae;g__Flavobacterium	4	32	0	2	4	33	0	0	7	3	0	0	5	2	0	27	0	3
p__Bacteroidota;c__Bacteroidia;o__Flavobacteriales;f__Weeksellaceae;g__Chryseobacterium	0	0	0	0	0	0	0	0	0	0	0	3	0	0	0	2	0	0
p__Bacteroidota;c__Bacteroidia;o__Flavobacteriales;f__Weeksellaceae;g__Cloacibacterium	0	0	2	0	5	0	4	0	0	0	0	0	0	6	0	12	0	0
p__Bacteroidota;c__Bacteroidia;o__Flavobacteriales;f__Weeksellaceae;g__Empedobacter	0	0	0	0	0	0	0	0	0	0	0	0	2	0	0	0	0	0
p__Bacteroidota;c__Bacteroidia;o__Sphingobacteriales;f__Sphingobacteriaceae;__	0	0	238	0	0	0	0	0	0	0	0	0	0	0	0	0	0	0
p__Bacteroidota;c__Bacteroidia;o__Sphingobacteriales;f__Sphingobacteriaceae;g__Pedobacter	0	5	146779	0	0	14	56	0	0	0	0	0	0	0	16	0	0	0
p__Bacteroidota;c__Bacteroidia;o__Sphingobacteriales;f__Sphingobacteriaceae;g__Sphingobacterium	0	0	0	0	0	0	20	0	0	0	17	28	3	0	0	0	0	48
p__Bdellovibrionota;c__Bdellovibrionia;o__Bacteriovoracales;f__Bacteriovoracaceae;g__Peredibacter	0	7	0	0	8	0	0	0	0	3	0	2	3	0	0	0	0	0
p__Bdellovibrionota;c__Bdellovibrionia;o__Bdellovibrionales;f__Bdellovibrionaceae;g__Bdellovibrio	0	5	0	0	0	0	0	0	0	0	0	0	0	0	0	9	0	0
p__Chloroflexi;c__Chloroflexia;o__Thermomicrobiales;f__JG30-KF-CM45;g__JG30-KF-CM45	0	0	0	0	0	0	0	0	0	5	4	0	0	0	0	0	0	0
p__Cyanobacteria;c__Cyanobacteriia;o__Chloroplast;f__Chloroplast;g__Chloroplast	5393	19	1350	23	145	1307	2512	241	363	466	276	285	495	816	165	384	83	409
p__Deinococcota;c__Deinococci;o__Deinococcales;f__Deinococcaceae;g__Deinococcus	0	15	0	0	0	0	0	0	0	0	6	3	0	23	0	40	0	0
p__Firmicutes;c__Bacilli;o__Bacillales;f__Bacillaceae;g__Anaerobacillus	0	6	0	0	0	0	0	0	0	0	0	0	0	0	0	0	0	0
p__Firmicutes;c__Bacilli;o__Bacillales;f__Bacillaceae;g__Bacillus	0	0	0	1218	0	0	6	0	0	0	0	0	0	0	0	2	0	0
p__Firmicutes;c__Bacilli;o__Bacillales;f__Bacillaceae;g__Terribacillus	0	0	0	0	0	0	0	0	0	0	0	9	0	0	0	0	0	0
p__Firmicutes;c__Bacilli;o__Bacillales;f__Bacillaceae;g__Virgibacillus	0	0	0	0	0	0	0	0	0	0	0	2	0	0	0	0	0	0
p__Firmicutes;c__Bacilli;o__Bacillales;f__Planococcaceae;g__Chryseomicrobium	0	0	0	0	0	0	0	0	0	5	5	0	0	0	0	12	0	0
p__Firmicutes;c__Bacilli;o__Bacillales;f__Planococcaceae;g__Lysinibacillus	0	0	0	0	0	0	4	0	0	0	0	0	0	0	0	0	0	0
p__Firmicutes;c__Bacilli;o__Bacillales;f__Planococcaceae;g__Psychrobacillus	86901	0	20	331246	34	45	0	0	0	0	0	24	0	0	0	0	0	0
p__Firmicutes;c__Bacilli;o__Exiguobacterales;f__Exiguobacteraceae;g__Exiguobacterium	0	0	0	0	0	0	0	0	0	0	0	0	0	0	0	0	0	4
p__Firmicutes;c__Bacilli;o__Lactobacillales;__;__	0	0	0	0	0	0	0	0	0	2	0	0	0	0	0	0	0	0
p__Firmicutes;c__Bacilli;o__Lactobacillales;f__Carnobacteriaceae;g__Granulicatella	0	0	0	0	0	0	0	0	0	0	5	0	0	0	0	0	0	0
p__Firmicutes;c__Bacilli;o__Lactobacillales;f__Carnobacteriaceae;g__Marinilactibacillus	2	0	6	0	8	229	0	0	0	0	6	6	0	0	0	4	4	2
p__Firmicutes;c__Bacilli;o__Lactobacillales;f__Enterococcaceae;g__Enterococcus	0	0	0	0	0	0	0	0	0	0	0	0	0	0	0	0	0	2
p__Firmicutes;c__Bacilli;o__Lactobacillales;f__Enterococcaceae;g__Tetragenococcus	0	0	0	0	9	0	0	0	0	0	5	0	5	0	0	0	0	0
p__Firmicutes;c__Bacilli;o__Lactobacillales;f__Listeriaceae;g__Brochothrix	0	0	0	0	0	0	0	0	0	0	2	0	0	0	0	0	0	0
p__Firmicutes;c__Bacilli;o__Lactobacillales;f__Streptococcaceae;g__Lactococcus	0	0	0	0	0	43	0	0	0	0	0	0	0	0	0	0	0	0
p__Firmicutes;c__Bacilli;o__Lactobacillales;f__Streptococcaceae;g__Streptococcus	0	0	0	0	0	0	0	0	0	0	10	0	4	0	0	0	0	0
p__Firmicutes;c__Bacilli;o__Paenibacillales;f__Paenibacillaceae;g__Paenibacillus	25	0	0	32739	103224	0	0	0	5	0	0	0	0	0	0	0	0	0
p__Firmicutes;c__Bacilli;o__Staphylococcales;f__Gemellaceae;g__Gemella	0	0	0	0	0	40	0	0	0	0	0	0	0	0	0	0	0	0
p__Firmicutes;c__Bacilli;o__Staphylococcales;f__Staphylococcaceae;g__Staphylococcus	0	8	3	0	9	114	0	5	3	0	22	3	0	7	3	21	7	7
p__Firmicutes;c__Clostridia;o__Peptostreptococcales-Tissierellales;f__Peptostreptococcales-Tissierellales;g__Tissierella	0	0	0	0	0	21	0	0	0	0	0	0	0	0	0	0	0	0
p__Firmicutes;c__Negativicutes;o__Veillonellales-Selenomonadales;f__Veillonellaceae;g__Veillonella	0	0	0	0	0	0	0	0	0	0	2	0	0	0	0	0	0	0
p__Fusobacteriota;c__Fusobacteriia;o__Fusobacteriales;f__Fusobacteriaceae;g__Fusobacterium	0	0	0	0	0	80	0	0	0	0	0	0	0	0	0	0	0	0
p__Gemmatimonadota;c__Longimicrobia;o__Longimicrobiales;f__Longimicrobiaceae;g__Longimicrobiaceae	0	0	0	0	0	0	0	0	0	0	0	2	0	0	0	0	0	0
p__Patescibacteria;c__Parcubacteria;o__Candidatus_Kaiserbacteria;f__Candidatus_Kaiserbacteria;g__Candidatus_Kaiserbacteria	0	0	0	0	7	42	0	0	0	0	0	0	0	0	0	0	0	0
p__Patescibacteria;c__Saccharimonadia;o__Saccharimonadales;f__LWQ8;g__LWQ8	0	6	0	0	0	0	3	2	0	0	0	0	6	0	0	8	0	0
p__Patescibacteria;c__Saccharimonadia;o__Saccharimonadales;f__Saccharimonadaceae;g__Candidatus_Saccharimonas	0	0	0	0	0	0	0	0	0	0	0	5	0	0	0	0	0	0

p__Patescibacteria;c__Saccharimonadia;o__Saccharimonadales;f__Saccharimonadaceae;g__TM7a	0	0	0	0	0	33	0	0	0	0	0	0	0	0	0	0	0	0
p__Patescibacteria;c__Saccharimonadia;o__Saccharimonadales;f__Saccharimonadaceae;g__TM7x	0	0	0	0	0	0	0	0	0	0	0	7	0	0	0	0	0	0
p__Patescibacteria;c__Saccharimonadia;o__Saccharimonadales;f__Saccharimonadales;g__Saccharimonadales	0	4	0	0	0	82	0	4	0	0	0	0	5	0	0	10	0	4
p__Planctomycetota;c__Planctomycetes;o__Pirellulales;f__Pirellulaceae;g__Pirellula	0	0	0	0	0	0	0	0	0	0	0	0	0	0	5	0	0	0
p__Planctomycetota;c__Planctomycetes;o__Pirellulales;f__Pirellulaceae;g__Rhodopirellula	0	0	0	0	0	0	0	0	0	0	0	0	0	0	3	0	0	0
p__Proteobacteria;__;__;__;__	0	0	0	0	0	0	0	0	0	0	0	0	0	0	0	0	0	2
p__Proteobacteria;c__Alphaproteobacteria;o__Acetobacterales;f__Acetobacteraceae;g__Acetobacteraceae	0	4	0	0	0	0	0	0	0	0	0	0	0	4	0	0	0	0
p__Proteobacteria;c__Alphaproteobacteria;o__Acetobacterales;f__Acetobacteraceae;g__Acidiphilium	0	0	0	0	0	0	0	0	0	0	0	0	7	0	0	0	0	0
p__Proteobacteria;c__Alphaproteobacteria;o__Acetobacterales;f__Acetobacteraceae;g__Roseomonas	0	0	0	0	0	0	10	0	0	0	0	0	11	0	0	6	0	0
p__Proteobacteria;c__Alphaproteobacteria;o__Azospirillales;f__Azospirillaceae;__	0	15	0	0	0	0	0	0	4	0	0	0	0	0	0	0	0	0
p__Proteobacteria;c__Alphaproteobacteria;o__Azospirillales;f__Azospirillaceae;g__Azospirillaceae	0	0	0	0	0	120	0	0	0	0	0	0	5	0	0	0	0	0
p__Proteobacteria;c__Alphaproteobacteria;o__Azospirillales;f__Azospirillaceae;g__Azospirillum	8	0	0	0	21	159	0	0	0	0	0	0	0	0	0	31	0	3
p__Proteobacteria;c__Alphaproteobacteria;o__Azospirillales;f__Azospirillaceae;g__Skermanella	0	0	0	0	0	0	0	0	0	0	0	0	0	0	0	0	1425	0
p__Proteobacteria;c__Alphaproteobacteria;o__Caulobacterales;f__Caulobacteraceae;__	0	53	0	0	0	126	13	0	12	0	0	4	6	6	0	98	0	0
p__Proteobacteria;c__Alphaproteobacteria;o__Caulobacterales;f__Caulobacteraceae;g__Brevundimonas	125	671	91	76	290	3241	162	83	143	111	124	201	183	159	193	880	48	133
p__Proteobacteria;c__Alphaproteobacteria;o__Caulobacterales;f__Caulobacteraceae;g__Caulobacter	20	268	18	13	42	429	33	16	24	20	43	25	63	46	12	357	0	16
p__Proteobacteria;c__Alphaproteobacteria;o__Caulobacterales;f__Caulobacteraceae;g__PMMR1	0	0	9	0	0	11	0	0	0	0	0	0	0	0	0	16	0	0
p__Proteobacteria;c__Alphaproteobacteria;o__Caulobacterales;f__Caulobacteraceae;g__Phenylobacterium	0	15	0	5	6	183	0	0	8	0	0	13	11	4	0	61	4	6
p__Proteobacteria;c__Alphaproteobacteria;o__Caulobacterales;f__Caulobacteraceae;g__uncultured	0	0	0	0	0	100	0	0	0	0	0	0	0	0	0	24	0	0
p__Proteobacteria;c__Alphaproteobacteria;o__Micavibrionales;f__uncultured;g__uncultured	0	0	0	0	0	57	0	0	0	0	0	0	0	0	0	7	0	0
p__Proteobacteria;c__Alphaproteobacteria;o__Rhizobiales;f__Beijerinckiaceae;__	0	0	0	0	0	0	0	0	0	0	0	0	10	0	0	0	0	0
p__Proteobacteria;c__Alphaproteobacteria;o__Rhizobiales;f__Beijerinckiaceae;g__Methylobacterium-Methylorubrum	0	20	0	0	16	0	246	9	11	45	8	25	175	940	25	173	57	174
p__Proteobacteria;c__Alphaproteobacteria;o__Rhizobiales;f__Beijerinckiaceae;g__Microvirga	0	5	0	0	0	0	4	0	0	6	0	0	11	0	0	24	0	0
p__Proteobacteria;c__Alphaproteobacteria;o__Rhizobiales;f__Beijerinckiaceae;g__uncultured	0	0	0	0	6	59	0	0	0	0	0	0	0	0	0	0	5	0
p__Proteobacteria;c__Alphaproteobacteria;o__Rhizobiales;f__Devosiaceae;g__Devosia	0	51	0	6	0	130	14	0	4	0	11	15	10	12	0	78	4	0
p__Proteobacteria;c__Alphaproteobacteria;o__Rhizobiales;f__Pleomorphomonadaceae;g__Chthonobacter	4	30	6	0	17	191	6	8	9	0	0	0	0	7	0	59	0	0
p__Proteobacteria;c__Alphaproteobacteria;o__Rhizobiales;f__Rhizobiaceae;__	0	55	0	12	26	319	0	0	0	0	0	0	0	0	0	6	0	0
p__Proteobacteria;c__Alphaproteobacteria;o__Rhizobiales;f__Rhizobiaceae;g__Allorhizobium-Neorhizobium-Pararhizobium-Rhizobium	8	58	0	6	40	399	832	0	0	12	57	183	20	26	39	133	45161	136
p__Proteobacteria;c__Alphaproteobacteria;o__Rhizobiales;f__Rhizobiaceae;g__Aureimonas	8	12	0	0	10	301	78	15	18	0	104	11	46	103	12	116	24	63
p__Proteobacteria;c__Alphaproteobacteria;o__Rhizobiales;f__Stappiaceae;g__Pannonibacter	7	138	0	11	17	208	13	6	10	8	4	12	10	15	0	179	0	7
p__Proteobacteria;c__Alphaproteobacteria;o__Rhizobiales;f__Xanthobacteraceae;g__Azorhizobium	0	11	0	0	7	0	0	0	0	0	0	3	0	0	0	0	0	0
p__Proteobacteria;c__Alphaproteobacteria;o__Rhodobacterales;f__Rhodobacteraceae;__	0	0	0	0	0	0	14	0	0	0	0	0	0	0	0	0	0	0
p__Proteobacteria;c__Alphaproteobacteria;o__Rhodobacterales;f__Rhodobacteraceae;g__Cereibacter	3	0	7	0	23	0	10	0	6	4	14	0	19	0	7	0	4	4
p__Proteobacteria;c__Alphaproteobacteria;o__Rhodobacterales;f__Rhodobacteraceae;g__Falsirhodobacter	0	0	0	9	0	0	9	0	0	0	0	0	0	0	0	0	0	0
p__Proteobacteria;c__Alphaproteobacteria;o__Rhodobacterales;f__Rhodobacteraceae;g__Paracoccus	4	33	11	0	15	260	0	5	0	0	0	0	6	0	0	62	0	5
p__Proteobacteria;c__Alphaproteobacteria;o__Rhodobacterales;f__Rhodobacteraceae;g__Rubellimicrobium	5	0	0	0	10	150	0	0	0	2	13	0	7	0	6	9	0	0
p__Proteobacteria;c__Alphaproteobacteria;o__Rhodospirillales;f__Rhodospirillaceae;g__Novispirillum	0	13	0	0	0	0	0	0	0	0	0	0	0	0	0	3	0	0
p__Proteobacteria;c__Alphaproteobacteria;o__Rickettsiales;f__Mitochondria;g__Mitochondria	174	0	125	0	12	44	294	23	31	40	15	8	50	143	24	42	4	35
p__Proteobacteria;c__Alphaproteobacteria;o__Sphingomonadales;f__Sphingomonadaceae;__	0	0	0	0	0	61	0	0	0	0	0	0	0	0	0	11	0	0
p__Proteobacteria;c__Alphaproteobacteria;o__Sphingomonadales;f__Sphingomonadaceae;g__Novosphingobium	0	4	0	3	0	0	0	0	0	0	31	0	0	89	0	0	14329	0
p__Proteobacteria;c__Alphaproteobacteria;o__Sphingomonadales;f__Sphingomonadaceae;g__Qipengyuania	0	17	0	0	12	155	0	0	0	0	0	0	9	0	0	37	0	0
p__Proteobacteria;c__Alphaproteobacteria;o__Sphingomonadales;f__Sphingomonadaceae;g__Sphingoaurantiacus	0	0	0	0	0	0	0	0	0	0	0	0	0	0	0	7	0	0
p__Proteobacteria;c__Alphaproteobacteria;o__Sphingomonadales;f__Sphingomonadaceae;g__Sphingobium	0	0	0	0	0	38	0	0	0	0	0	0	0	10	0	6	252	0
p__Proteobacteria;c__Alphaproteobacteria;o__Sphingomonadales;f__Sphingomonadaceae;g__Sphingomonas	16	33	17	0	44	567	194	0	25	37	36	38	64	70	6	94	179	78
p__Proteobacteria;c__Alphaproteobacteria;o__Sphingomonadales;f__Sphingomonadaceae;g__Sphingopyxis	0	0	0	0	0	44	0	0	0	0	0	0	0	0	0	0	0	0
p__Proteobacteria;c__Gammaproteobacteria;o__Aeromonadales;f__Aeromonadaceae;g__Aeromonas	9	5	8	5	45	54	0	0	10	3	0	5	3	6	0	0	5	6
p__Proteobacteria;c__Gammaproteobacteria;o__Alteromonadales;f__Alteromonadaceae;g__Rheinheimera	0	13	0	0	0	218	8	0	0	0	4	0	0	0	0	26	0	6
p__Proteobacteria;c__Gammaproteobacteria;o__Alteromonadales;f__Shewanellaceae;g__Shewanella	5	9	6	5	48	221	7	0	7	0	19	0	26	0	12	15	9	14
p__Proteobacteria;c__Gammaproteobacteria;o__Burkholderiales;f__Alcaligenaceae;__	0	0	0	0	0	0	0	0	0	0	0	0	0	0	0	0	1427	0
p__Proteobacteria;c__Gammaproteobacteria;o__Burkholderiales;f__Alcaligenaceae;g__Pigmentiphaga	0	0	0	0	0	0	0	0	0	0	0	0	0	0	0	0	6579	0
p__Proteobacteria;c__Gammaproteobacteria;o__Burkholderiales;f__Alcaligenaceae;g__Verticiella	0	715	4	113	0	0	0	0	0	0	0	3	0	441	40	0	27529	37
p__Proteobacteria;c__Gammaproteobacteria;o__Burkholderiales;f__Burkholderiaceae;g__Burkholderia-Caballeronia-Paraburkholderia	0	0	0	0	0	109	0	0	0	0	0	2	0	3	0	0	0	0
p__Proteobacteria;c__Gammaproteobacteria;o__Burkholderiales;f__Burkholderiaceae;g__Cupriavidus	0	65	0	0	19	217	7	2	5	0	6	0	4	0	3	58	0	0
p__Proteobacteria;c__Gammaproteobacteria;o__Burkholderiales;f__Burkholderiaceae;g__Lautropia	0	5	0	0	0	0	0	0	0	0	0	0	0	0	0	4	0	0
p__Proteobacteria;c__Gammaproteobacteria;o__Burkholderiales;f__Burkholderiaceae;g__Limnobacter	0	8	0	0	0	0	0	0	0	0	0	0	0	0	0	0	0	0
p__Proteobacteria;c__Gammaproteobacteria;o__Burkholderiales;f__Chromobacteriaceae;g__Vogesella	0	0	0	0	6	0	0	0	0	0	0	0	9	0	0	0	0	0
p__Proteobacteria;c__Gammaproteobacteria;o__Burkholderiales;f__Comamonadaceae;__	22	105	0	17	62	740	13	0	24	12	29	24	26	11	12	154	8	14
p__Proteobacteria;c__Gammaproteobacteria;o__Burkholderiales;f__Comamonadaceae;g__Acidovorax	0	0	0	0	0	0	3	0	4	0	5	0	0	0	6	0	0	0
p__Proteobacteria;c__Gammaproteobacteria;o__Burkholderiales;f__Comamonadaceae;g__Aquabacterium	0	137	0	9	0	168	4	19	13	4	0	12	9	26	7	180	0	18
p__Proteobacteria;c__Gammaproteobacteria;o__Burkholderiales;f__Comamonadaceae;g__Comamonas	22	30	8	0	54	427	13	11	25	23	23	19	46	18	25	59	5	16
p__Proteobacteria;c__Gammaproteobacteria;o__Burkholderiales;f__Comamonadaceae;g__Diaphorobacter	0	11	0	0	0	0	0	0	0	0	0	0	0	0	0	17	0	0
p__Proteobacteria;c__Gammaproteobacteria;o__Burkholderiales;f__Comamonadaceae;g__Pelomonas	0	0	0	0	0	46	0	0	0	0	0	0	0	0	0	0	0	0
p__Proteobacteria;c__Gammaproteobacteria;o__Burkholderiales;f__Neisseriaceae;g__Conchiformibius	0	0	0	0	0	0	0	0	0	0	0	0	0	0	0	0	2	2
p__Proteobacteria;c__Gammaproteobacteria;o__Burkholderiales;f__Nitrosomonadaceae;g__Nitrosomonas	0	0	0	0	0	0	3	0	0	0	0	0	0	0	0	0	0	0
p__Proteobacteria;c__Gammaproteobacteria;o__Burkholderiales;f__Oxalobacteraceae;g__Massilia	70	183	25320	0	277	2504	36	44	33	44	77	1117	102	57	13	239	18803	28
p__Proteobacteria;c__Gammaproteobacteria;o__Burkholderiales;f__Oxalobacteraceae;g__Noviherbaspirillum	22	202	9	15	60	784	24	12	23	18	31	13	40	31	17	356	8	12
p__Proteobacteria;c__Gammaproteobacteria;o__Cardiobacteriales;f__Cardiobacteriaceae;g__uncultured	0	0	5	0	3	0	0	0	0	0	0	0	0	0	0	0	0	0
p__Proteobacteria;c__Gammaproteobacteria;o__Cellvibrionales;f__Cellvibrionaceae;g__Cellvibrio	0	0	0	0	0	0	0	0	0	0	0	0	0	0	0	2	0	0
p__Proteobacteria;c__Gammaproteobacteria;o__Enterobacterales;__;__	0	0	0	0	0	0	0	0	0	0	0	0	0	41	0	0	1195	0
p__Proteobacteria;c__Gammaproteobacteria;o__Enterobacterales;f__Enterobacteriaceae;__	0	0	0	0	0	0	0	0	0	0	2	0	0	23	0	0	0	3162
p__Proteobacteria;c__Gammaproteobacteria;o__Enterobacterales;f__Enterobacteriaceae;g__Enterobacter	0	0	0	0	0	0	0	0	0	0	0	0	0	23	0	0	0	0
p__Proteobacteria;c__Gammaproteobacteria;o__Enterobacterales;f__Enterobacteriaceae;g__Escherichia-Shigella	0	10	0	0	5	133	0	0	0	0	0	0	0	0	0	0	0	0

p__Proteobacteria;c__Gammaproteobacteria;o__Enterobacterales;f__Enterobacteriaceae;g__Kosakonia	49	30	0	0	36	598	0	0	0	45	0	131	31	0	5	0	31	6
p__Proteobacteria;c__Gammaproteobacteria;o__Enterobacterales;f__Erwiniaceae;__	0	0	0	0	0	0	32	0	39	27	51	54	1702	274	129	657	5	1902
p__Proteobacteria;c__Gammaproteobacteria;o__Enterobacterales;f__Erwiniaceae;g__Pantoea	1978	110	65	123	107	276	200371	154278	149852	188521	245779	235687	127139	202062	232242	92680	64649	158736
p__Proteobacteria;c__Gammaproteobacteria;o__Enterobacterales;f__Yersiniaceae;g__Serratia	3	0	6	0	0	137	0	0	0	0	0	0	0	0	0	0	0	0
p__Proteobacteria;c__Gammaproteobacteria;o__Pasteurellales;f__Pasteurellaceae;g__Haemophilus	0	0	0	0	0	193	0	0	0	0	0	0	0	0	0	0	0	0
p__Proteobacteria;c__Gammaproteobacteria;o__Pasteurellales;f__Pasteurellaceae;g__Pasteurella	0	0	5	0	0	0	0	0	0	0	0	0	0	0	0	0	0	0
p__Proteobacteria;c__Gammaproteobacteria;o__Pseudomonadales;f__Moraxellaceae;g__Acinetobacter	308	44	208	140	1047	3701	124	151	220	214	357	291	319	135	246	72	79	174
p__Proteobacteria;c__Gammaproteobacteria;o__Pseudomonadales;f__Moraxellaceae;g__Alkanindiges	9	0	13	3	63	237	0	13	0	13	13	0	9	10	0	6	0	9
p__Proteobacteria;c__Gammaproteobacteria;o__Pseudomonadales;f__Moraxellaceae;g__Enhydrobacter	8	0	0	0	6	173	3	3	0	0	0	5	0	0	0	0	3	2
p__Proteobacteria;c__Gammaproteobacteria;o__Pseudomonadales;f__Moraxellaceae;g__Moraxella	0	0	0	0	3	0	0	0	0	0	0	0	0	0	0	0	0	0
p__Proteobacteria;c__Gammaproteobacteria;o__Pseudomonadales;f__Moraxellaceae;g__Psychrobacter	5	0	0	0	8	0	0	0	0	0	0	0	0	0	0	0	0	5
p__Proteobacteria;c__Gammaproteobacteria;o__Pseudomonadales;f__Pseudomonadaceae;__	6	0	8	0	7	63	0	0	0	6	0	8	0	0	0	0	0	0
p__Proteobacteria;c__Gammaproteobacteria;o__Pseudomonadales;f__Pseudomonadaceae;g__Pseudomonas	443	112	54	47	126	1446	47301	23387	26939	17157	34620	26923	145632	81245	64235	129861	105742	145623
p__Proteobacteria;c__Gammaproteobacteria;o__Xanthomonadales;f__Xanthomonadaceae;g__Lysobacter	0	8	3	0	0	270	0	0	5	5	0	0	0	0	4	26	0	0
p__Proteobacteria;c__Gammaproteobacteria;o__Xanthomonadales;f__Xanthomonadaceae;g__Stenotrophomonas	0	20711	0	0	2	0	4	2	0	0	3	0	53	0	1220	18	38817	42
p__Proteobacteria;c__Gammaproteobacteria;o__Xanthomonadales;f__Xanthomonadaceae;g__Xanthomonas	0	82455	0	0	0	5	0	0	0	51	0	18	16	117	213	9	2789	29
p__Spirochaetota;c__Spirochaetia;o__Spirochaetales;f__Spirochaetaceae;g__Treponema	0	0	0	0	0	0	0	0	0	0	0	0	0	0	3	0	0	0
	95834	106657	288708	365846	106114	23152	252522	178347	177890	206945	281850	265247	276626	287021	298969	227511	329287	311086
