# Supplementary material for: Investigation of microorganisms in cannabis after heating in a commercial vaporizer
Source: Front Cell Infect Microbiol. 2023 Jan 13;12:1051272. doi: 10.3389/fcimb.2022.1051272 (PMC9880168; doi:10.3389/fcimb.2022.1051272)
Supplement: Supplementary file 3 [file Table_2.pdf]

#OTU ID	P_0	P_0	P_30	P_30	P_70	P_70	L_0	L_0	L_30	L_30	L_70	L_70	H_0	H_0	H_30	H_30	H_70	H_70
k__Fungi;__;__;__;__;__	35	0	517	36	203	0	221	343	0	65	1639	69	456	428	10	17	0	98
p__Ascomycota;__;__;__;__	18	0	0	0	0	0	0	0	0	0	6	0	0	0	0	0	0	0
p__Ascomycota;c__Dothideomycetes;__;__;__	0	0	0	0	0	0	0	0	0	0	0	47	0	0	0	0	0	0
p__Ascomycota;c__Dothideomycetes;o__Capnodiales;__;__	0	0	0	0	0	0	0	0	0	0	0	0	0	0	66	0	0	0
p__Ascomycota;c__Dothideomycetes;o__Capnodiales;f__Cladosporiaceae;g__Cladosporium	20	402	183	896	1562	0	2295	2626	1044	362	385	485	64	909	181	482	79	312
p__Ascomycota;c__Dothideomycetes;o__Capnodiales;f__Cladosporiaceae;g__Toxicocladosporium	0	0	0	0	0	0	0	283	0	0	0	0	0	0	0	0	10	0
p__Ascomycota;c__Dothideomycetes;o__Capnodiales;f__Mycosphaerellaceae;g__Mycosphaerella	0	0	0	0	0	0	102	0	0	0	0	0	95	158	0	43	0	0
p__Ascomycota;c__Dothideomycetes;o__Capnodiales;f__Mycosphaerellaceae;g__Septoria	0	0	0	0	0	0	218	26	0	0	0	0	0	0	0	0	0	0
p__Ascomycota;c__Dothideomycetes;o__Dothideales;__;__	0	0	0	0	0	68	0	0	0	0	0	0	0	0	0	0	0	0
p__Ascomycota;c__Dothideomycetes;o__Dothideales;f__Aureobasidiaceae;g__Kabatiella	0	0	0	0	0	0	79	887	404	216	0	56	0	0	0	62	0	0
p__Ascomycota;c__Dothideomycetes;o__Pleosporales;f__Didymellaceae;__	0	0	0	0	0	0	0	0	0	0	0	0	379	0	0	0	0	0
p__Ascomycota;c__Dothideomycetes;o__Pleosporales;f__Didymellaceae;g__Didymella	0	0	0	0	0	0	436	0	0	0	191	0	0	0	0	0	0	0
p__Ascomycota;c__Dothideomycetes;o__Pleosporales;f__Didymellaceae;g__Epicoccum	0	0	0	0	0	90	0	0	0	0	0	0	0	0	0	0	0	0
p__Ascomycota;c__Dothideomycetes;o__Pleosporales;f__Didymellaceae;g__Neodidymelliopsis	0	0	0	0	0	0	300	0	0	0	0	0	0	0	0	0	0	0
p__Ascomycota;c__Dothideomycetes;o__Pleosporales;f__Pleosporaceae;g__Alternaria	113	4376	106	5209	1812	2048	2081	1595	2010	1450	443	824	174	640	61	530	130050	383
p__Ascomycota;c__Dothideomycetes;o__Pleosporales;f__Pleosporaceae;g__Curvularia	0	0	0	0	0	0	0	0	0	39	0	0	0	0	0	44	0	0
p__Ascomycota;c__Dothideomycetes;o__Pleosporales;f__Pleosporaceae;g__Exserohilum	0	73	0	0	0	0	0	0	0	0	0	0	0	0	0	0	0	0
p__Ascomycota;c__Dothideomycetes;o__Pleosporales;f__Pleosporales_fam_Incertae_sedis;g__Paradictyoarthrinium	0	0	113	0	0	0	0	0	0	0	0	0	0	0	0	0	0	0
p__Ascomycota;c__Eurotiomycetes;o__Eurotiales;f__Aspergillaceae;g__Aspergillus	0	0	154	0	0	0	0	102	0	0	0	0	0	0	49	0	0	0
p__Ascomycota;c__Eurotiomycetes;o__Eurotiales;f__Aspergillaceae;g__Penicillium	0	267	615	108	73	0	76	424	0	111	0	0	107	0	285	33	0	0
p__Ascomycota;c__Leotiomycetes;o__Erysiphales;f__Erysiphaceae;g__Golovinomyces	0	0	0	0	0	0	0	0	0	0	69	0	0	0	0	0	0	0
p__Ascomycota;c__Leotiomycetes;o__Erysiphales;f__Erysiphaceae;g__Podosphaera	0	0	0	0	0	0	0	0	0	0	0	0	30	0	0	0	0	0
p__Ascomycota;c__Leotiomycetes;o__Thelebolales;f__Pseudeurotiaceae;g__Pseudogymnoascus	121420	11	0	0	10	0	0	0	0	0	0	125	0	0	0	0	0	0
p__Ascomycota;c__Pezizomycetes;o__Pezizales;f__Sarcosomataceae;g__Donadinia	0	0	0	0	0	0	0	0	0	0	0	0	0	36	0	0	0	0
p__Ascomycota;c__Saccharomycetes;o__Saccharomycetales;__;__	0	0	0	0	0	0	0	0	19	0	0	0	0	0	0	0	0	0
p__Ascomycota;c__Saccharomycetes;o__Saccharomycetales;f__Phaffomycetaceae;g__Wickerhamomyces	0	0	103	0	0	0	0	0	0	0	0	0	0	0	0	0	0	0
p__Ascomycota;c__Saccharomycetes;o__Saccharomycetales;f__Saccharomycetales_fam_Incertae_sedis;g__Candida	0	0	0	0	0	0	0	0	72	0	0	148	0	0	0	0	0	0
p__Ascomycota;c__Sordariomycetes;__;__;__	0	0	0	0	0	0	0	0	0	105	0	0	0	0	0	0	0	0
p__Ascomycota;c__Sordariomycetes;o__Diaporthales;f__Diaporthaceae;g__Diaporthe	0	0	0	0	0	0	0	14	0	0	0	0	0	914	0	0	158	210
p__Ascomycota;c__Sordariomycetes;o__Glomerellales;f__Glomerellaceae;g__Colletotrichum	0	0	0	0	0	0	58	0	0	0	0	0	0	0	0	0	0	0
p__Ascomycota;c__Sordariomycetes;o__Glomerellales;f__Plectosphaerellaceae;g__Gibellulopsis	0	0	0	0	0	0	0	0	0	0	0	0	0	0	0	0	1791	0
p__Ascomycota;c__Sordariomycetes;o__Glomerellales;f__Plectosphaerellaceae;g__Lectera	0	0	0	0	0	0	110	0	0	181	0	0	0	0	0	0	0	0
p__Ascomycota;c__Sordariomycetes;o__Glomerellales;f__Plectosphaerellaceae;g__Plectosphaerella	0	0	0	0	0	0	0	0	0	0	0	0	141	0	0	0	0	0
p__Ascomycota;c__Sordariomycetes;o__Hypoceales;f__Catabotrydaceae;g__unidentified	0	0	110	0	0	0	0	0	0	0	0	0	0	0	0	0	0	0
p__Ascomycota;c__Sordariomycetes;o__Hypocreales;f__Hypocreaceae;g__Trichoderma	0	0	0	0	41	0	0	0	0	0	0	0	0	0	0	0	0	0
p__Ascomycota;c__Sordariomycetes;o__Hypocreales;f__Hypocreales_fam_Incertae_sedis;g__Acremonium	0	0	62	0	0	0	0	0	0	0	0	90	0	0	241	0	0	390
p__Ascomycota;c__Sordariomycetes;o__Hypocreales;f__Hypocreales_fam_Incertae_sedis;g__Sarocladium	0	0	0	0	0	0	0	0	89	0	0	0	0	0	0	0	0	0
p__Ascomycota;c__Sordariomycetes;o__Hypocreales;f__Nectriaceae;g__Fusarium	0	0	0	0	0	0	20319	0	19990	0	0	0	0	0	0	0	0	0
p__Ascomycota;c__Sordariomycetes;o__Hypocreales;f__Nectriaceae;g__Gibberella	28	1863	306	1184	2437	156	75	1581	937	73	0	286	132	0	151	294	11	679
p__Ascomycota;c__Sordariomycetes;o__Hypocreales;f__Sarocladiaceae;g__Parasarocladium	0	0	0	0	0	0	8	0	0	0	0	0	0	0	0	0	0	0
p__Ascomycota;c__Sordariomycetes;o__Hypocreales;f__Stachybotryaceae;g__Stachybotrys	0	0	0	0	94	0	0	0	0	0	0	0	0	0	0	0	0	0
p__Ascomycota;c__Sordariomycetes;o__Trichosphaeriales;f__Trichosphaeriaceae;g__Nigrospora	0	0	0	0	0	0	0	0	0	0	0	0	0	0	49	0	0	0
p__Ascomycota;c__Sordariomycetes;o__Xylariales;f__Xylariaceae;g__Zygosporium	0	0	0	0	0	0	127	0	0	0	0	0	0	0	0	0	0	0
p__Basidiomycota;__;__;__;__	0	0	0	61	0	0	0	184	9	170	0	0	0	0	62	0	0	19
p__Basidiomycota;c__Agaricomycetes;o__Agaricales;f__Psathyrellaceae;g__Coprinellus	0	0	0	0	0	0	0	143	0	0	0	0	0	0	0	0	0	0
p__Basidiomycota;c__Agaricomycetes;o__Agaricales;f__Psathyrellaceae;g__Coprinopsis	0	0	0	0	0	0	0	0	0	0	0	0	0	1803	0	0	0	0
p__Basidiomycota;c__Agaricomycetes;o__Cantharellales;f__Ceratobasidiaceae;g__Thanatephorus	0	0	0	0	0	0	0	0	0	0	0	0	69	0	0	0	0	0
p__Basidiomycota;c__Agaricomycetes;o__Hymenochaetales;f__Schizoporaceae;g__Hyphodontia	0	0	0	0	0	0	0	0	0	0	0	0	0	0	0	76	0	0
p__Basidiomycota;c__Agaricomycetes;o__Phallales;f__Phallaceae;g__Phallus	0	0	0	0	61	0	0	0	0	0	0	0	0	0	0	0	0	0
p__Basidiomycota;c__Agaricomycetes;o__Polyporales;f__Ganodermataceae;g__Perenniporia	0	0	0	0	0	0	0	0	0	0	0	0	0	107	0	0	0	0
p__Basidiomycota;c__Agaricomycetes;o__Polyporales;f__Steccherinaceae;g__Physodontia	0	0	0	0	117	0	0	0	0	0	0	0	0	0	0	0	0	0
p__Basidiomycota;c__Agaricostilbomycetes;o__Agaricostilbales;f__Kondoaceae;g__Kondoa	0	0	0	0	0	0	0	0	0	0	0	0	0	64	0	0	0	0
p__Basidiomycota;c__Cystobasidiomycetes;o__Cystobasidiales;f__Cystobasidiaceae;g__Cystobasidium	5	0	0	0	0	0	457	0	0	0	309	0	0	0	0	0	9002	0
p__Basidiomycota;c__Cystobasidiomycetes;o__Cystobasidiales;f__Cystobasidiaceae;g__Occultifur	0	0	0	0	0	0	0	0	0	0	196	0	0	0	0	0	0	0
p__Basidiomycota;c__Cystobasidiomycetes;o__Cystobasidiomycetes_ord_Incertae_sedis;f__Symmetrosporaceae;g__Symmetrospora	13	2	0	0	0	0	1546	159	1346	379	0	519	611	2101	670	913	13889	605
p__Basidiomycota;c__Exobasidiomycetes;o__Golubeviales;f__Golubeviaceae;g__Golubevia	4	0	0	0	0	0	0	0	0	0	0	0	0	0	0	0	0	0
p__Basidiomycota;c__Malasseziomycetes;o__Malasseziales;f__Malasseziaceae;g__Malassezia	48	972	218	464	844	199	156	776	159	832	240	521	124	451	283	29	3	266
p__Basidiomycota;c__Malasseziomycetes;o__Malasseziales;f__Malasseziaceae;g__unidentified	0	0	0	0	63	0	0	0	0	0	0	0	0	0	0	0	0	0
p__Basidiomycota;c__Microbotryomycetes;o__Sporidiobolales;f__Sporidiobolaceae;g__Rhodotorula	0	0	0	0	0	0	0	0	44	0	0	0	74	0	0	0	0	0
p__Basidiomycota;c__Tremellomycetes;__;__;__	0	0	0	0	0	0	0	0	0	0	0	0	0	317	412	0	0	512
p__Basidiomycota;c__Tremellomycetes;o__Filobasidiales;f__Filobasidiaceae;g__Filobasidium	21	5	0	0	81	0	7991	7941	9939	1647	8552	6357	720	1013	69	536	0	1250
p__Basidiomycota;c__Tremellomycetes;o__Filobasidiales;f__Filobasidiaceae;g__Naganishia	0	0	0	0	0	0	0	0	60	0	0	49	0	0	0	0	0	158
p__Basidiomycota;c__Tremellomycetes;o__Tremellales;f__Bulleribasidiaceae;g__Dioszegia	0	0	0	0	0	0	141	0	0	0	0	0	0	0	0	0	0	0
p__Basidiomycota;c__Tremellomycetes;o__Tremellales;f__Bulleribasidiaceae;g__Hannaella	8	0	0	7	0	4	0	0	151	10	97	0	0	53444	1780	0	0	300
p__Basidiomycota;c__Tremellomycetes;o__Tremellales;f__Bulleribasidiaceae;g__Vishniacozyma	0	0	0	3765	0	0	0	0	0	0	1304	71	47	0	0	0	0	0
p__Basidiomycota;c__Tremellomycetes;o__Tremellales;f__Rhynchogastremataceae;g__Papiliotrema	0	0	0	0	0	0	0	0	0	0	0	0	0	0	0	0	548	77
p__Basidiomycota;c__Tremellomycetes;o__Tremellales;f__Tremellales_fam_Incertae_sedis;g__Kwoniella	0	0	0	0	0	0	0	0	0	0	0	0	134	0	0	0	0	0
p__Basidiomycota;c__Tremellomycetes;o__Tremellales;f__Trimorphomycetaceae;g__Saitozyma	0	0	0	0	0	0	0	0	0	0	0	0	0	11	0	0	0	0
p__Basidiomycota;c__Ustilaginomycetes;o__Ustilaginales;f__Ustilaginaceae;__	0	0	0	0	0	0	0	0	0	0	0	0	0	31	0	7	0	0
p__Basidiomycota;c__Ustilaginomycetes;o__Ustilaginales;f__Ustilaginaceae;g__Moesziomyces	0	0	0	0	0	0	63	0	0	0	0	41	0	89	0	0	0	0
p__Basidiomycota;c__Wallemiomycetes;o__Wallemiales;f__Wallemiaceae;g__Wallemia	0	0	0	0	0	0	0	160	0	0	0	0	0	0	0	0	0	0
	121733	7971	2487	11730	7398	2565	36859	17244	36273	5640	13431	9688	3357	62516	4369	3066	155541	5259
